# Supplementary material for: Novel Selective PPARα Modulator Pemafibrate for Dyslipidemia, Nonalcoholic Fatty Liver Disease (NAFLD), and Atherosclerosis
Source: Metabolites. 2023 May 2;13(5):626. doi: 10.3390/metabo13050626 (PMC10221566; doi:10.3390/metabo13050626)
Supplement: Supplementary file 1 [file metabolites-13-00626-s001.zip › metabolites-2233327-supplementary.pdf]

Table S1. Effects of Pemafibrate on Dyslipidemia

	References
Basic	
β-oxidation↑, VLDL secretion↓, TG clearance↑, LPL activity↑, VLDL receptor↑	[35, 38, 39]
NPC1L1↓	[39, 40]
FGF21↑	[35, 39, 44]
ABCA1↑, ABCG1↑	[37]
Clinical	
TG↓, HDL-C↑, RemL-C↓, nonHDL-C↓, apoB→↓, apoB-48↓, apoC-III↓	[47, 53, 54, 59, 67, 69, 70, 75, 112]
Non-fasting TG↓, Non-fasting RemL-C↓, Non-fasting apoB-48↓	[53, 54]
Small LDL particle number↓, Small HDL particle number↑	[56]
Cholesterol efflux capacity↑, HDL_3-C↑, pre β1HDL↑, apoA-1↑	[53]

Abbreviations: VLDL, very-low-density lipoprotein; TG, triglyceride; LPL, lipoprotein lipase; FGF21, fibroblast growth factor 21; HDL-C, high-density lipoprotein cholesterol; RemL-C, remnant lipoprotein cholesterol; apo, apolipoprotein.

Table S2. Effects of Pemafibrate on NAFLD/NASH

	References
Basic	
Fatty liver→↓, Ballooning↓, Inflammation↓, Fibrosis↓	[84–86]
Steatohepatitis↓, Hepatic steatosis↓, Cardiac dysfunction↓	[87]
Clinical	
ALT↓, ALP↓, γ-GT↓, AST/platelet ratio index↓, FIB-4 index↓ (although there are some variations)	[88–93]
ALT↓, saturated fatty acid↓, unsaturated fatty acid↓	[94]
PEMA-FL (MRI-PDFF→, MRE↓, ALT↓, serum liver fibrosis markers↓)	[95]
PROMINENT (Safety outcome for NAFLD: HR, 0.78; 95%CI, 0.63-0.96)	[110]

Abbreviations: NAFLD, non-alcoholic fatty liver disease; NASH, non-alcoholic steatohepatitis; ALT, alanine aminotransferase; ALP, alkaline phosphatase; γ-GT, γ-glutamyl transpeptidase; AST, aspartate aminotransferase; MRI-PDFF, MRI-estimated proton density fat fraction; MRE, magnetic resonance elastography; HR, hazard ratio; CI, confidence interval.

Table S3. Effects of Pemafibrate on Atherosclerosis and ASCVD

	References
Basic	
Revascularization in hindlimb ischemia ↑, phosphorylation of eNOS ↑, FGF21 ↑	[100]
Acetylcholine-induced endothelial relaxation rates ↑ (combination with pitavastatin), phosphorylation of eNOS ↑	[101]
Vasoconstrictive eicosanoids and free fatty acids ↓	[102]
Mean ratio of Macrophages to plaque area ↓	[103]
VSMC proliferation ↓, Neointima formation after vascular injury ↓	[104]
Coronary stent-induced cellular inflammation ↓, Neointimal hyperplasia ↓	[105]
Atherosclerotic lesions ↓, F4/80 ↓, VCAM1 ↓, IL-6 ↓	[37]
Development and inflammation of vein graft lesions ↓	[106]
Neutrophil adhesion on atheroprone femoral artery ↓	[107]
AAA rupture ↓	[108]
Clinical	
PROMINENT (Nonfatal MI, ischemic stroke, coronary revascularization, or cardiovascular death: HR, 1.03; 95%CI, 0.91-1.15)	[110]

Abbreviations: ASCVD, atherosclerotic cardiovascular diseases; eNOS, endothelial nitric oxide synthase; FGF21, fibroblast growth factor 21; VSMC, vascular smooth muscle cells; AAA, abdominal aortic aneurysm; MI, myocardial infarction; HR, hazard ratio; CI, confidence interval.
